# Signaling Pathways That Mediate Alveolar Macrophage Activation by Surfactant Protein A and IL-4

**DOI:** 10.3389/fimmu.2022.860262

**Published:** 2022-04-04

**Authors:** Belén García-Fojeda, Carlos M. Minutti, Carlos Montero-Fernández, Cordula Stamme, Cristina Casals

**Affiliations:** ^1^ Department of Biochemistry and Molecular Biology, Complutense University of Madrid, Madrid, Spain; ^2^ Division of Cellular Pneumology, Research Center Borstel, Leibniz Lung Center, Borstel, Germany; ^3^ Department of Anesthesiology and Intensive Care, University of Lübeck, Lübeck, Germany

**Keywords:** IL-4, surfactant protein A, macrophage alternative activation, proliferation, Pi3k-akt, mTORC1, PKCζ, metabolism

## Abstract

Activation of tissue repair program in macrophages requires the integration of IL-4/IL-13 cytokines and tissue-specific signals. In the lung, surfactant protein A (SP-A) is a tissue factor that amplifies IL-4Rα-dependent alternative activation and proliferation of alveolar macrophages (AMs) through the myosin18A receptor. However, the mechanism by which SP-A and IL-4 synergistically increase activation and proliferation of AMs is unknown. Here we show that SP-A amplifies IL-4-mediated phosphorylation of STAT6 and Akt by binding to myosin18A. Blocking PI3K activity or the myosin18A receptor abrogates SP-A´s amplifying effects on IL-4 signaling. SP-A alone activates Akt, mTORC1, and PKCζ and inactivates GSK3α/β by phosphorylation, but it cannot activate arginase-1 activity or AM proliferation on its own. The combined effects of IL-4 and SP-A on the mTORC1 and GSK3 branches of PI3K-Akt signaling contribute to increased AM proliferation and alternative activation, as revealed by pharmacological inhibition of Akt (inhibitor VIII) and mTORC1 (rapamycin and torin). On the other hand, the IL-4+SP-A-driven PKCζ signaling axis appears to intersect PI3K activation with STAT6 phosphorylation to achieve more efficient alternative activation of AMs. Consistent with IL-4+SP-A-driven activation of mTORC1 and mTORC2, both agonists synergistically increased mitochondrial respiration and glycolysis in AMs, which are necessary for production of energy and metabolic intermediates for proliferation and alternative activation. We conclude that SP-A signaling in AMs activates PI3K-dependent branched pathways that amplify IL-4 actions on cell proliferation and the acquisition of AM effector functions.

## Introduction

Macrophages are crucial regulators of the initiation, maintenance, and resolution of repair following injury (1, 2). Tissue repair activities of macrophages are associated with type II cytokine activation (IL-4 and IL-13) through IL-4Rα. Global and myeloid cell-specific disruption of the IL-4Rα gene has been shown to cause tissue repair deficiencies in the lung and liver in different models of injury (3, 4). Mechanistically, it has been proposed that IL-4Rα-activation of macrophages induces, either directly or through other cell types, the secretion of growth factors like IGF-1 and the production of repair factors like i) collagen type I, alpha 1, which forms the extracellular matrix; ii) resistin-like molecule alpha (Relmα), which serves to cross-link collagen with fibrils; and iii) arginine-derived polyamines, which are necessary for collagen synthesis and cell proliferation (1, 2, 5). Furthermore, IL-4Rα signaling instructs macrophage proliferation, resulting in local expansion of this effector population (6).

We recently reported that IL-4 may not be sufficient for the full induction of alternative activation and proliferation of tissue macrophages and that involvement of local tissue signals is also required (4). Surfactant protein (SP-) A, in the lung, and the first component of the complement system (C1q), in the liver, enhance IL-4Rα-dependent alternative activation, proliferation, and tissue-repair functions of resident macrophages through binding their collagen domains to the myosin 18A receptor (4). SP-A and C1q are versatile recognition proteins, which belong to the group of secreted soluble defense collagens that includes C1q, collectins (e.g., SP-A, SP-D, mannan-binding lectin), ficolins, and adiponectin (7). SP-A is secreted to the alveolar fluid by epithelial type II cells. This protein has important functions in the lung, integrating intrinsic host-defense properties in a lipid-rich medium (pulmonary surfactant) required to protect the lung against alveolar collapse during the breathing cycle (7–9). Thus, SP-A performs its host defense functions and at the same time facilitates the surface-active biophysical functions of the surfactant system (10).

SP-A significantly boosted IL-4-mediated proliferation and alternative activation induced by IL-4 in human, mouse, and rat alveolar macrophages (AMs) (4). Mice lacking SP-A exhibited reduced IL-4-dependent activation and proliferation of AMs. Interestingly, IL-4 increases the production of SP-A by epithelial type II cells and the expression of myosin 18A receptor (Myo18A) on the surface of AMs for full IL-4-dependent alternative activation and proliferation (4). Studies with SP-A-deficient mice infected with the lung-migrating nematode *Nippostrongylus brasiliensis* demonstrated that SP-A is required for i) accelerating parasite clearance; ii) reducing pulmonary injury after infection; and iii) promoting tissue repair (4).

In this study we asked what are the intracellular signaling pathways by which SP‐A amplifies IL‐4‐effects in AMs. IL-4 signals through both the type I receptor (expressed on myeloid cells and lymphocytes and composed of the IL-4Rα chain and IL-2Rγ chain, also called γc) and the type II receptor (expressed on myeloid cells and all non-hematopoietic cells and composed of the IL-4Rα and IL-13Rα1 chains). Both IL-4 receptors signal *via* the Janus kinase (JAK)/signal transducer and activator of transcription 6 (STAT6) pathway that induces robust expression of genes associated with alternative activation in bone marrow-derived macrophages (BMDM) and peritoneal macrophages (pMs) (11, 12). IL-4 activation of the type I receptor also induces highly efficient tyrosine phosphorylation of insulin receptor substrate 2 (IRS2). Although IRS2 does not possess intrinsic enzymatic activities, it functions as an activator for a few other signaling proteins such as phosphatidylinositol-3 kinase (PI3K). Thus, IRS2 triggers the PI3K/Akt/mTORC1 pathway that activates cell growth, proliferation, and synthesis of extracellular matrix macromolecules in BMDM and pMs (12–15). PI3K/Akt activation also enhances the expression of a subset of alternative activation markers, such as *Arg1* and *Retnla* in BMDM (13, 14) and pMs (15). Given that SP-A activates the PI3K signal transduction pathway to upregulate the mannose receptor expression in human monocyte derived macrophages (16) and promote atypical protein kinase C ζ (PKCζ) phosphorylation (17) and regulation of endolysosomal trafficking dependent on the PI3K/PKCζ axis (18), we hypothesized that the induction of PI3K signaling by SP-A could increase IL-4 actions on AMs.

The objective of this study was to dissect SP-A-dependent signaling pathways that strengthen IL-4 signaling in AMs and to determine the metabolic profile of these cells. Our results indicate that SP-A enhances IL-4 effects in AMs through activation of PI3K, Akt, mTORC1, and PKCζ, and inactivation of GSK3α/β. In addition, we found that SP-A and IL-4 act synergistically to increase mitochondrial respiration and glycolysis. These processes provide energy and metabolic intermediates that sustain alternative activation and proliferation of alveolar macrophages.

## Materials and Methods

### Reagents

Cell culture media and reagents were from Lonza (Basel, Switzerland). Western blotting materials were obtained from Bio-Rad (Hercules, California). Akt inhibitor VIII and PKCζ pseudosubstrate inhibitor myristoylated were from Calbiochem, (Darmstadt, Germany). PI3K inhibitor (LY294002), rapamycin and torin-1, as well as antibodies against phospho-STAT6 (Tyr641), phospho-Akt (Ser 473), phospho-PKCζ/λ (Thr410/403), phospho-4E-BP1 (Ser65), phospho-GSK3β (Ser 9), phospho-FoxO3a (Thr32), their respective total protein antibodies, and α-tubulin were purchased at Cell Signaling Technologies (Danvers, Massachusetts). Antibodies against anti-β actin, anti-PKCζ H1 and anti-EEA1 were from Santa Cruz Biotechnology (Dallas, Texas), and anti-STAT6, Alexa Fluor 488 anti-mouse IgG, and Alexa Fluor 633 anti-rabbit IgG were from Thermo Fisher Scientific (Waltham, Massachusetts). All other reagents were of analytical grade, purchased from Sigma-Aldrich (St. Louis, Missouri) unless otherwise specified.

### Isolation, Purification and Characterization of Native SP-A

Surfactant protein A was isolated from bronchoalveolar lavages (BAL) of patients with alveolar proteinosis using a sequential butanol and octylglucoside extraction (4, 19–23). The purity of SP-A was checked by one-dimensional SDS-PAGE in 12% acrylamide under reducing conditions and mass spectrometry. The oligomerization state of SP-A was assessed by electrophoresis under non-denaturing conditions (22, 23), electron microscopy (23), and analytical ultracentrifugation as reported elsewhere (22). SP-A consisted of supratrimeric oligomers of at least 18 subunits. Each subunit had a relative molecular mass (Mr) 36 kDa. The endotoxin content of native or recombinant human SP-A was < 0.1 endotoxin units/mg of SP-A as determined by Limulus amebocyte lysate assay (Lonza).

### Isolation and Culture of Primary AMs

AMs were obtained from BAL of male Sprague–Dawley rats (Envigo). Rats (∼350 g) were anesthetized with ketamine (80 mg/kg; Merial, Duluth, GA) and xylazine (10 mg/kg; Bayer, Leverkusen, Germany) and the cardiopulmonary block was extracted to perform BALs with 40ml of PBS (0.2mM EDTA). Animal handling procedures to obtain BAL were reviewed and approved by the local ethics committee (both Complutense University of Madrid and Autonomous Community of Madrid), according to Directive 2010/63/EU of the European Parliament and the Spanish law RD53/2013 on protection of animals used for experimentation. In some experiments, AMs were obtained from BAL of SP-A-deficient mice as previously reported (4, 24). Importantly, AMs from SP-A-deficient mice were normal in number, phenotype and ability to respond to IL-4 (4). Animal care was conducted according to the Schleswig-Holstein Ministry of Energy, Agriculture, the Environment, Nature and Digitalization. Mice were housed under pathogen-free conditions with an inverted 12-hour light/dark cycle and had free access to food and water.

Bronchoalveolar cells were separated from lavage fluid by centrifugation (250 g, 7 min). The sedimented cells were washed twice with PBS, and the cell pellet was resuspended in RPMI 1640 medium (10% heat inactivated FBS, 100 U/ml penicillin, and 100 mg/ml streptomycin, supplemented with 2 mM glutamine). AMs were purified by adherence for 90 min at 37°C under a 95% air-5% CO_2_ atmosphere in 150-mm culture dishes as reported previously (4, 19, 24). Adherent cells were 94.0 ± 1.1% viable (trypan blue exclusion test). To evaluate the purity of the isolated macrophages, rat AMs were immuno-stained with anti-CD11c (AbD Serotec, Kidlington, U.K.) and were analyzed by flow cytometry. Adherent cells were found to be composed of 90 ± 1% AMs.

### Incubation Conditions

Adherent cells were gently scraped, plated in 96-well plastic dishes (7.5 x 10^4^ cells/well) in 0.2 ml RPMI 1640 medium supplemented with 5% FBS, 2 mM glutamine, 100 U/ml penicillin, and 100 mg/ml streptomycin, and pre-cultured overnight. Cells were cultured in the presence or absence of rat recombinant IL-4 (1 μg/ml) (ImmunoTools, Berlin, Germany) and/or SP-A (25, 50 and 100 μg/ml) as reported previously (4). SP-A was added simultaneously with IL-4 to cells. Lower doses of IL-4 (0.1-1 μg/ml) were also assayed. Different concentrations of inhibitors were titrated to minimize undesired effects and toxicity. After titration the following concentrations were used: 50 nM LY294002 (PI3K inhibitor), 25 or 100 nM Akt inhibitor VIII (Isozyme-selective, Akti-1/2) as indicated, 30 μM PKCζ Pseudosubstrate inhibitor, myristoylated, 25 or 100 nM torin-1, and 5 or 20 nM rapamycin, as indicated. Cell viability was 95 or 97% under assay conditions and after treatment with inhibitors. Macrophage cultures were plated in triplicate wells, and each series of experiments was repeated at least three times.

### siRNA-Targeted Silencing of Myo18A

After isolation, primary AMs were resuspended in Amaxa^®^ mouse macrophage nucleofector solution (Lonza) and nucleofected with 100 nM siRNA using a nucleofector 2b device (Lonza) as previously reported (4). Experiments were conducted using two Stealth siRNAs directed against rat Myo18A (RSS322720 and RSS322721) (Applied Biosystems, Carlsbad, California). Medium GC Stealth siRNA was used as control (12935300) (Applied Biosystems). Myo18A expression was detected by Western blot analysis with an anti-Myo18A antibody as reported previously (4). After 48 hours post nucleofection, Myo18A expression was reduced 72 ± 4% for RSS322720 and 71 ± 5% for RSS322721 compared to control. At this time-point, cells were stimulated.

### Immunoprecipitation of PKCζ

AMs were stimulated with SP-A, IL-4 or combinations thereof for 8 minutes. PKCζ immunoprecipitation was performed as in (17), with modifications. After culture, cells were lysed at 4°C for 30 min in 500 μl of lysis buffer: 50 mM Tris-HCl (pH 8.0), 150 mM NaCl, 0.5 mM EDTA, 0.5% IGEPAL, 1 mM benzamidine, 200 µg/ml aprotinin, 200 μg/ml leupeptin, 1 mM phenylmethylsulfonyl fluoride (PMSF), 20 mM β-glycerophosphate, 10 mM NaF, 10 mM sodium pyrophosphate and 2 mM orthovanadate. The lysates were centrifuged at 10 000 × g for 10 min, and the supernatants were precleared by adding protein A-agarose (50 μl) and incubated at 4°C for 45 min, followed by centrifugation at 10 000 × g for 10 min. The precleared supernatant was incubated with anti-PKCζ antibody or control IgG at 4°C overnight, after which 50 μl of protein A-agarose was added for 3 h at 4°C with gentle rotation. The immune complexes were collected by centrifugation at 10,000 × g for 5 min at 4°C, washed three times with cold lysis buffer, and released by boiling with Laemmli loading buffer. Phosphorylation of PKCζ was subsequently analyzed by Western blot using an anti-Phospho-PKCζ/λ (Thr410/403) and anti-PKCζ, as described below.

### Western Blot Analysis

AMs were stimulated with SP-A, IL-4 or combinations thereof for 90 min to determine p-STAT6(Tyr641), 30 min to analyze p-Akt (Ser473) and p-4E-BP1 (Ser65), 10 min for p-GSK3 α/β (Ser21/9), and 45 min for p-FoxO3a (Thr32). Cells were lysed by shaking 30 min at 4°C with a buffer containing: 10 mM HEPES (pH 7.9), 15 mM MgCl_2_, 10 mM KCl, 0.5 mM EDTA, 0.2% Triton X-100, 1 mM benzamidine, 200 µg/ml aprotinin, 200 µg/mL leupeptin, 1 mM phenylmethylsulfonyl fluoride (PMSF), 20 mM β-glycerophosphate, 10 mM NaF, 10 mM sodium pyrophosphate and 2 mM orthovanadate (Sigma-Aldrich). Samples were resolved by SDS-PAGE under reducing conditions and transferred to polyvinylidene fluoride membranes (Bio-Rad). After blocking with 2.5% (m/v) skim milk, membranes were washed in PBS 0.1% Tween20 and incubated with anti-phospho or anti-total protein antibodies, as indicated, overnight at 4°C. The membranes were washed, incubated with HRP-labeled anti-rabbit/mouse IgG, and exposed to ECL reagents (Merck Millipore, Darmstadt, Germany). Immunoreactive bands intensity were quantified (Quantity One Software; Bio-Rad), and then normalized to the respective total protein for quantification of phosphorylated proteins, except p-4E-BP1 which was normalized relative to β-Actin.

### Arginase Activity Assay

Arginase activity was measured as previously reported (4). Activated AMs with or without IL-4 and/or SP-A at 37°C for 48h were lysed with 50 μl of 50 mM Tris–HCl (pH 7.5), 0.1% Triton X-100, 1 mM benzamidine, 200 μg/ml aprotinin, and 200 μg/ml leupeptin. After 30 min shaking at 4°C, arginase was activated with 50 μl of 10 mM MnCl_2_ and 50 mM Tris-HCl, pH 7.5, for 10 min at 55°C. L-arginine hydrolysis was measured by incubating the cell lysate with 25 μl of 0.5 M L-arginine (Sigma-Aldrich) (pH 9.7) at 37°C for 1 h. The reaction was stopped by addition of 200 μl H_2_SO_4_/H_3_PO_4_/H_2_O (1:3:7 v/v). The produced urea was quantified at 570 nm after addition of 25 μl of α-isonitrosopropiophenone (dissolved in 100% ethanol) followed by heating at 99°C for 45 min. Urea production was normalized to cell number for each treatment by quantifying cells with the WST-1 reagent (Roche), following manufacturer’ instructions. One unit of arginase activity is defined as the amount of enzyme that catalyses the formation of 1 μmol urea per min.

### Cell Proliferation Assays

For 5-Bromo-2’-deoxyuridine (BrdU) incorporation analysis (4), cells were treated with IL-4 and/or SP-A for 24h. Then, cells were exposed to 10 µM BrdU for another 24h. Detection of BrdU incorporation was performed by ELISA using BrdU cell proliferation assay (Cell Signaling Technologies) as previously reported (4). Briefly, cells are fixed, and DNA is denatured. Then, a BrdU mouse monoclonal antibody is added to detect the incorporated BrdU, followed by anti-mouse IgG linked to HRP secondary antibody. Finally, the HRP substrate tetramethylbenzidine is added to develop color. The retention of the cells at the bottom of the well after the removal of the supernatant was checked by the WST-1 assay. Cell number in all treatments was similar before and after supernatant removal.

### Bioenergetic Characterization of AMs

AMs were plated at 2 x 10^5^ cells/well in 1-, 5- or 11-mM glucose RPMI, during 24 h in a poly-L-lysine-coated Seahorse XFe24 microplate. RPMI was supplemented with 2 mM glutamine, 5% heat-inactivated FBS and 100 U or μg/ml of penicillin/streptomycin. Then, macrophages were stimulated with SP-A, IL-4 or combinations thereof for 24 h. Afterwards, cells were incubated in bicarbonate-free RPMI supplemented with glucose (1-, 5- or 11-mM), 2mM glutamine, and 2% FBS, at 37°C in a CO2-free incubator. Oxygen consumption rate (OCR) and extracellular acidification rate (ECAR) were simultaneously measured in a Seahorse XFe24 Extracellular Flux Analyzer (Agilent Technologies, CA). After four measurements under basal conditions, cells were treated sequentially with 1 μM oligomycin, 0.6 μM carbonyl cyanide p-(trifluoromethoxy) phenylhydrazone (FCCP), 0.4 μM FCCP, and 1 μM rotenone plus 1 μM antimycin A, with three consecutive determinations under each condition. Nonmitochondrial respiration (OCR value after addition of rotenone plus antimycin A) was subtracted from all OCR measurements. Spare respiratory capacity was calculated by subtracting basal OCR from the maximal respiratory capacity, which is the maximal OCR in the presence of FCCP. Glycolytic reserve was calculated by subtracting basal ECAR from the ECAR rate obtained after oligomycin treatment.

### Glucose Uptake Assays

Glucose uptake was measured as previously reported (25). Briefly, AMs were plated at 2 x 10^5^ cells/well in 5mM glucose RPMI (supplemented with 2 mM glutamine, 5% heat-inactivated FBS and 100 U or μg/ml of penicillin/streptomycin) in a 24-well plate. The next day, cells were stimulated with SP-A, IL-4 or combinations thereof for 24 h. Then, the fluorescent glucose analogue, 2-deoxy-2-[(7-nitro-2,1,3-benzoxadiazol-4-yl) amino]-D-glucose (2-NBDG) was added to the wells at a concentration of 50 μg/ml and cells were incubated for 20 minutes at 37°C under a 95% air-5% CO2 atmosphere. The 2-NBDG uptake reaction was stopped by removing the incubation medium and washing the cells twice with PBS. Fluorescence was measured by flow cytometry using a FACScalibur (Becton Dickinson, New Jersey) flow cytometer and Cell Quest software. Background fluorescence was measured in control cells without 2-NBDG.

### Confocal Microscopy/PKCζ Membrane Translocation

AMs from SP-A-deficient mice were used to avoid confounding effects of the endogenous protein. Macrophages were seeded at 1x10^5^ cells/well on 8-well Lab-TekII chamber slides (Nunc, Wiesbaden, Germany) and allowed to adhere for 90 min at 37°C in a 5% CO_2_ atmosphere. After treatment, the cells were fixed with ice-cold (-20°C) methanol, washed with PBS, followed by permeabilization with 0.25% Triton X-100. Subsequently, the cells were blocked with 10% BSA/PBS, washed and incubated with anti-PKCζ H1 and rabbit anti-EEA1 antibodies (1:250 and 1:60, respectively, Santa Cruz). Alexa Fluor 488 anti-mouse IgG and Alexa Fluor 633 anti-rabbit IgG (1:500, Thermo Fisher Scientific) was used as secondary antibody. Cell nuclei were counterstained with DAPI (Thermo Fisher Scientific). Samples were analyzed using a Leica TCS SP5 confocal laser scanning microscope (Leica Microsystems, Wetzlar, Germany). Confocal images were acquired with the Leica Application Suite AF software and analysis was performed with the Sync Windows Plugin for Image J.

### Statistics

Statistical evaluation of different groups was performed by analysis of variance (ANOVA) followed by the Bonferroni multiple comparison test. An α level ≤ 5% (p ≤ 0.05) was considered significant. All statistical calculations were performed using PRISM (Graphpad La Jolla, CA).

## Results

### SP-A Enhances IL-4 Signaling Through the Myo18A Receptor

**Figure 1:**
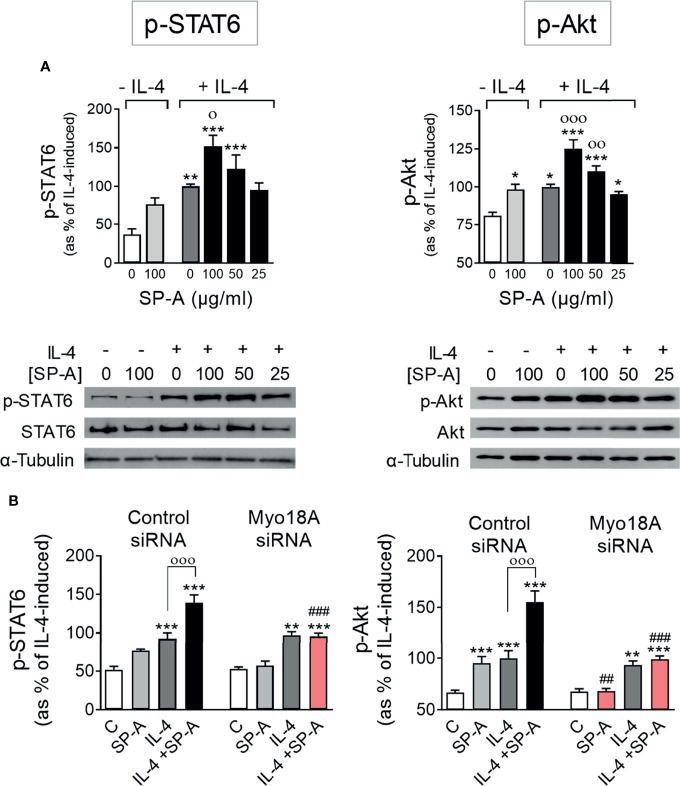
SP-A acts through Myo18A to enhance STAT6 and Akt activation elicited by IL-4 in AMs. **(A)** Purified rat AMs were treated with or without IL-4 (1 μg/ml) in the presence or absence of SP-A at the indicated concentrations. STAT6 and Akt phosphorylation relative to total STAT6 or Akt, respectively, were analyzed by Western blot. A representative Western blot for p-STAT6 (Tyr641) or p-Akt (Ser473) is shown below each graph. **(B)** AMs were nucleofected with Myo18A (RSS322720) or control siRNA. Cells were then stimulated with or without IL-4 (1 μg/ml) and/or SP-A (100 μg/ml), and p-STAT6 and p-Akt were analyzed by Western blot. The data shown are means ± SEM of three different AM cultures with at least three biological replicates. ANOVA followed by the Bonferroni multiple-comparison test was used. *p < 0.05, **p < 0.01, and *** < 0.001 when compared with untreated cells. ^о^ *p* < 0.05, ^оо^ *p* < 0.01, and ^ооо^ *p* < 0.001 when SP-A+IL-4-treated macrophages were compared with IL-4-treated macrophages. ^##^p < 0.01 and ^###^ *p* < 0.001 when the effect of Myo18A siRNA is compared with siRNA control.

First, we determined whether SP-A could drive IL-4-mediated activation of STAT6 and Akt. These are the two signaling branches that are triggered in response to IL-4 type I receptor activation and are essential for alternative activation and proliferation of macrophages (6, 11, 12). **Figure 1A** shows the phosphorylation of STAT6 (Tyr 641) and Akt (Ser 473) induced by IL-4 stimulation of AMs. Phosphorylation of STAT6 and Akt was potentiated by SP-A in a dose-dependent manner. In the absence of IL-4, SP-A could induce significant phosphorylation of Akt, but not STAT6, confirming the observation that SP-A induces activation of the PI3K-Akt axis in AMs (16–18).

To analyze whether the Myo18A receptor is mediating SP-A signaling in alveolar macrophages, we silenced the Myo18A gene and studied the phosphorylation of STAT6 and Akt in IL-4-stimulated macrophages in the presence or absence of SP-A. Silencing Myo18A resulted in a reduction of its expression by 72 ± 4% for RSS322720 siRNA and 71 ± 5% for RSS322721 siRNA compared to control (**Supplementary Figure 1**). **Figure 1B** shows that silencing the Myo18A receptor abrogated the effects of SP-A on STAT6 and Akt phosphorylation in macrophages stimulated with IL-4. In the absence of IL-4, we observed that silencing the Myo18A receptor also abolished Akt phosphorylation induced by SP-A (**Figure 1B**), indicating that SP-A induces PI3K/Akt activation by binding to the Myo18A receptor. However, Myo18A silencing had no impact on p-STAT6 and p-Akt induced by IL-4 alone, in the absence of SP-A. Together, these data suggest that the binding of SP-A to the Myo18A receptor amplifies IL-4-mediated phosphorylation of STAT6 and Akt. In addition, our data demonstrate that IL-4R signaling does not require Myo18A and therefore the synergy between IL-4 and SP-A must occur at the level of intracellular signaling.

### SP-A Amplification of IL-4 Actions Depends on PI3K Activation

**Figure 2:**
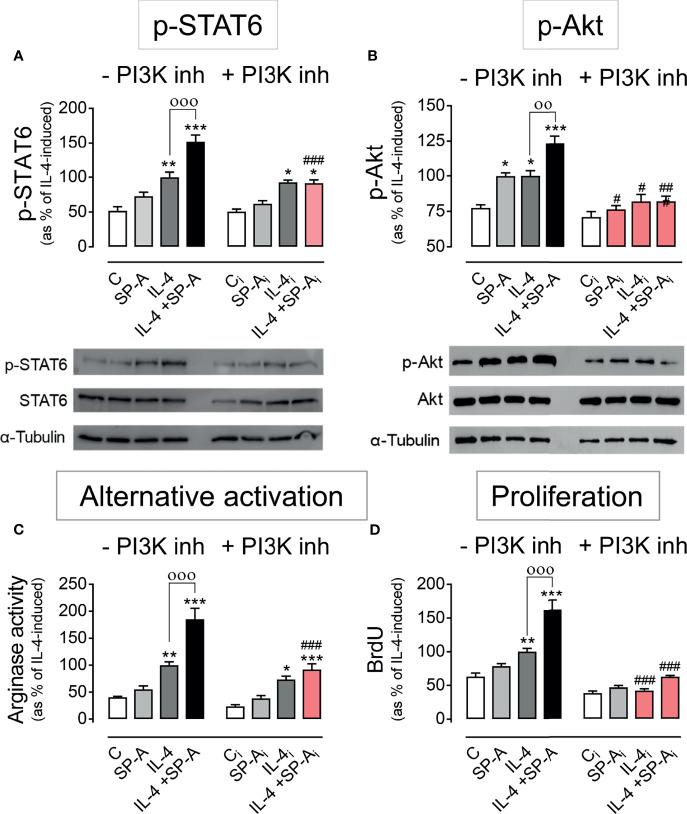
PI3K inhibition abrogates SP-A effects on IL-4 signaling. Purified rat AMs were pretreated with 50 nM LY294002 (PI3K inhibitor) or vehicle (DMSO) for two hours. Subsequently, cells were stimulated with or without IL-4 (1 μg/ml) in the presence or absence of SP-A (100 μg/ml) and exposed to 10 μM BrdU for proliferation analysis. Quantification of **(A)** p-STAT6 (Tyr641) relative to total STAT6, **(B)** p-Akt (Ser473) relative to total Akt, **(C)** arginase activity, and **(D)** BrdU incorporation in the newly synthesized DNA of AMs. A representative Western blot for p-STAT6 and p-Akt is shown below each graph. The results are presented as means (± SEM) from three different cell cultures with at least three biological replicates. ANOVA followed by the Bonferroni multiple-comparison test was used. *p < 0.05, **p < 0.01, and ***p < 0.001 when compared with untreated macrophages. ^оо^ *p* < 0.01 and ^ооо^ *p* < 0.001 when SP-A+IL-4-treated macrophages were compared with IL-4-treated macrophages. ^#^ *p* < 0.05, ^##^p < 0.01 and ^###^ *p* < 0.001 when the effect of PI3K inhibitor is compared with the same sample without inhibitor.

To assess if SP-A amplification of IL-4 actions depends on PI3K activation, we inhibited PI3K with 50 nM LY294002 in IL-4-stimulated macrophages with or without SP-A and analyzed the phosphorylation of STAT6 (**Figure 2A**) and Akt (**Figure 2B**), as well as alternative activation of AM by measuring arginase activity (**Figure 2C**), and proliferation by analyzing BrdU incorporation in DNA (**Figure 2D**). As expected, IL-4 did depend on PI3K to induce p-Akt, but not STAT6 phosphorylation. However, PI3K inhibitor abrogated SP-A-driven enhancement of both STAT6 and Akt phosphorylation in IL4-stimulated cells. The inhibition of PI3K nullified SP-A-driven enhancement of alternative activation and proliferation of IL4-stimulated alveolar macrophages (**Figures 2C, D**). However, only proliferation, but not alternative activation, was PI3K-dependent in macrophages treated with IL-4 alone. This is consistent with the fact that IL-4R signals *via* the JAK/STAT6 pathway induces robust alternative activation. Taken together, these results indicate that SP-A activates Myo18A/PI3K-dependent coordinated signaling pathways that amplify IL-4 actions.

### Akt Activation Is Essential for SP-A and IL-4-Mediated Macrophage Proliferation

**Figure 3:**
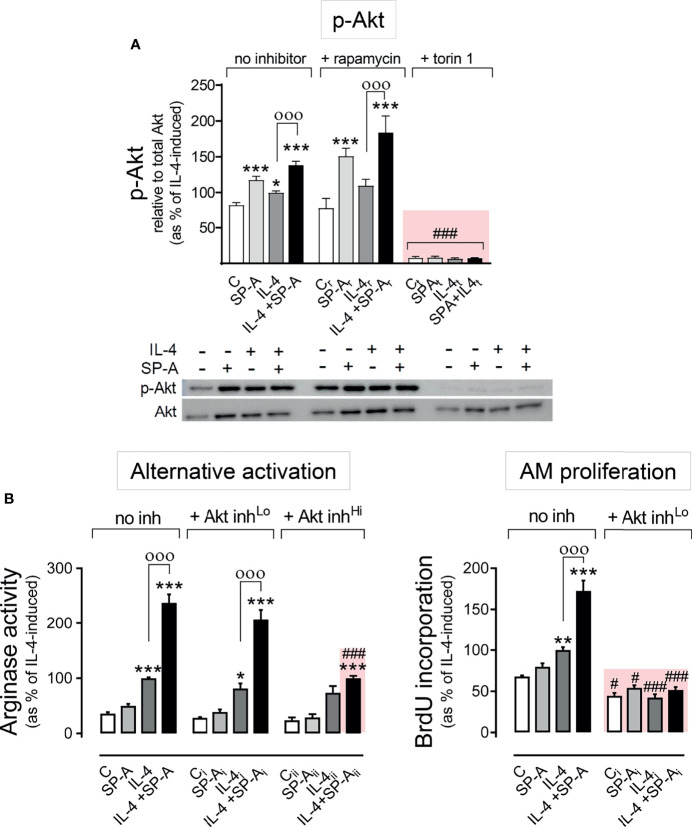
SP-A and IL-4 induce Akt activation to enhance alternative activation and proliferation of AMs. **(A)** Purified rat AMs were pre-treated with 20 nM rapamycin, 100 nM torin 1, or vehicle for one hour. Cells were then stimulated with or without IL-4 (1 μg/ml) in the presence or absence of SP-A (100 μg/ml) 30 minutes, then Western blot for quantification of p-Akt (Ser473) relative to total Akt was performed. A representative Western blot image is shown below the graph. In **(B)** arginase activity and BrdU incorporation were measured in purified rat AMs pretreated with 25 nM (Lo), 100 nM (Hi) Akt inhibitor VIII, or vehicle for two hours, and then stimulated with or without IL-4 (1 μg/ml) and/or SP-A (100 μg/ml). The results are presented as means (± SEM) from three different cell cultures with at least three biological replicates. ANOVA followed by the Bonferroni multiple-comparison test was used. *p < 0.05, **p < 0.01, and ***p < 0.001 when compared with untreated macrophages. ^ооо^ *p* < 0.001 when SP-A+IL-4-treated macrophages were compared with IL-4-treated macrophages. ^#^ *p* < 0.05 and ^###^ *p* < 0.001 when the effect of any inhibitor (Akt inhibitor VIII or torin1) is compared with the same sample without inhibitor.

One of the best-characterized targets of PI3K is the Akt/mTORC signaling pathway (26). Upon PI3K activation, Akt is phosphorylated by PDK1 at Thr 308 and by mTORC2 at Ser 473. Maximal activation of Akt requires phosphorylation of Ser473 at the hydrophobic motif. We next evaluated whether the phosphorylation of Akt at Ser 473 induced by SP-A, IL-4, or both was dependent on mTORC2. **Figure 3A** shows that the increase in p-Akt (Ser473) elicited by SP-A, IL-4, and SP-A+IL-4 was completely suppressed by torin (inhibitor of mTORC1 and mTORC2) but not by rapamycin (specific inhibitor of mTORC1), confirming that the complex responsible for phosphorylation of Akt at Ser473 was mTORC2.

To evaluate whether Akt affects the proliferation and alternative activation of alveolar macrophages stimulated with IL-4 and/or SP-A, we inhibited Akt activation with the Akt inhibitor VIII at concentrations of 25 or 100 nM (**Figure 3B**). We observed that 25 nM Akt inhibitor VIII drastically suppressed AM proliferation by IL-4 alone, which confirmed previous observations (15), and by IL-4 in combination with SP-A. In contrast, low concentrations of Akt inhibitor VIII did not affect arginase activity measured in alveolar macrophages stimulated with IL-4 or IL-4+SP-A (**Figure 3B**). Higher concentrations of Akt inhibitor VIII are needed to block arginase activity stimulated by IL-4+SP-A. These results demonstrate that while Akt activation is essential for macrophage proliferation, other signaling pathways, in addition to the PI3K/Akt pathway, are directly involved in signaling that promotes alternative activation of alveolar macrophages.

### SP-A and IL-4 Inactivate GSK3α/β by Akt-Dependent Phosphorylation and Enhance mTORC1 Kinase Activity

**Figure 4:**
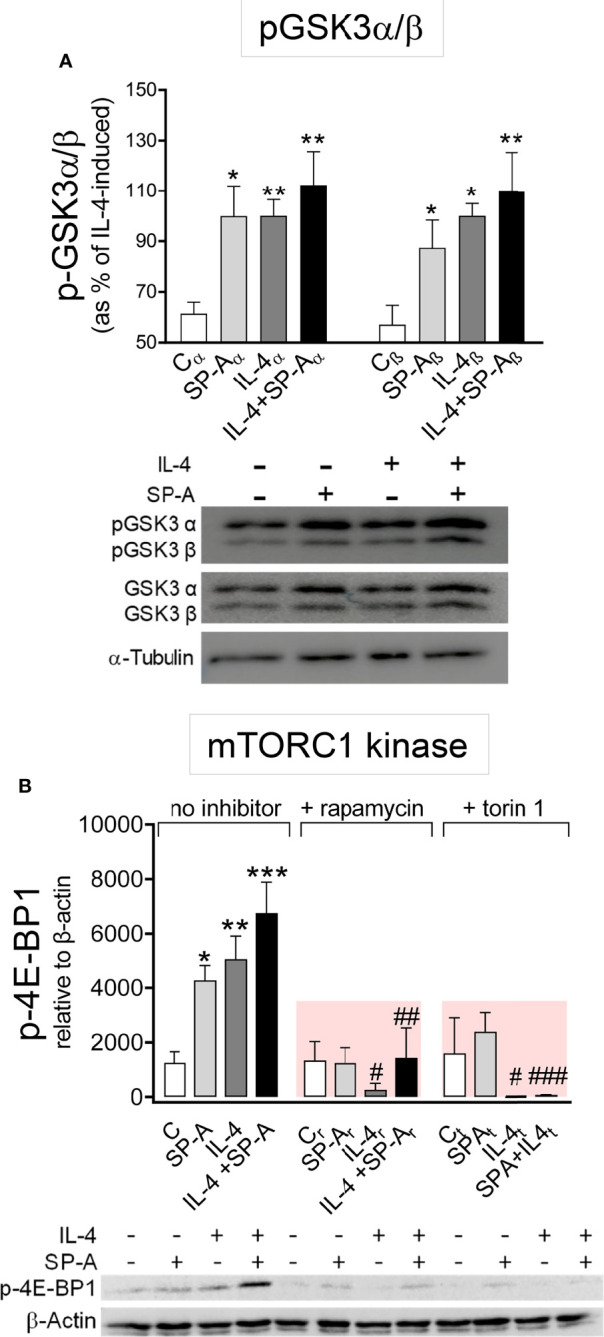
SP-A and IL-4 enhance GSK3α/β phosphorylation and mTORC1 activation. **(A)** AMs were stimulated with or without IL-4 (1μg/ml) and/or SP-A (100 μg/ml) and phosphorylation of GSK3-α/β(Ser21/9) was measured. **(B)** Purified rat AMs were pretreated with 20 nM rapamycin, 100 nM torin 1, or vehicle (no inhibitor) for one hour. Cells were then stimulated with or without IL-4 (0.5 μg/ml) in the presence or absence of SP-A (40 μg/ml). p-4E-BP1(Ser65) relative to β-actin was quantified by Western blot. Representative Western blot analyses are shown below each graph. The results are presented as means (± SEM) from three different AM cultures with at least three biological replicates. ANOVA followed by the Bonferroni multiple-comparison test was used. *p < 0.05, **p < 0.01, and ***p < 0.001 when compared with untreated macrophages. ^#^ *p* < 0.05, ^##^ *p* < 0.01, and ^###^ *p* < 0.001 when the effect of either rapamycin or torin1 is compared with the same sample without inhibitor.

The three best-established downstream targets of Akt are glycogen synthase kinase 3 (GSK3), forkhead box O family of transcription factors (FoxO), and mTORC 1 (26). We first analyzed whether IL-4 and/or SP-A affected the phosphorylation of GSK3αβ and FoxO3a in alveolar macrophages. Akt-dependent phosphorylation of the α and β subunits of GSK3 blocks the inhibitory actions of GSK3 on metabolism, proliferation, or cell survival (26). **Figure 4A** shows that SP-A, IL-4, and IL-4+SP-A (no additive effect) significantly increased phosphorylation of GSK3α and β subunits (in the residues Ser 21 and 9, respectively), which is consistent with the role of IL-4 and IL-4+SP-A in increasing macrophage proliferation. Inhibition of Akt suppressed GSK3 phosphorylation (data not shown). Regarding FoxO3a, a gene transcriptional activator also known as a tumor suppressor, we found that FoxO3a phosphorylation was not affected by IL-4 and/or SP-A stimulation of alveolar macrophages (**Supplementary Figure 2**).

We next analyzed whether IL-4 and/or SP-A affected mTORC1 activation, which promotes anabolic processes, cell growth, and proliferation (26) and enhances the expression of a subset of alternative activation markers in BMDM (13, 14). One of the main targets of mTORC1 activation is eukaryotic translation initiation factor 4E-binding protein 1 (4E-BP1), which is used as a canonical mTORC1 substrate. We evaluated phosphorylation of 4E-BP1 (**Figure 4B**) and found that the kinase activity of mTORC1 increased in the presence of SP-A, IL-4, and IL-4+SP-A. The activity of mTORC1 kinase was completely inhibited by rapamycin and torin 1 (**Figure 4B**).

### Pharmacological Inhibition of mTORC1 suppresses IL-4- and IL-4+SP-A-Mediated Alternative Activation and Proliferation of Alveolar Macrophages

**Figure 5:**
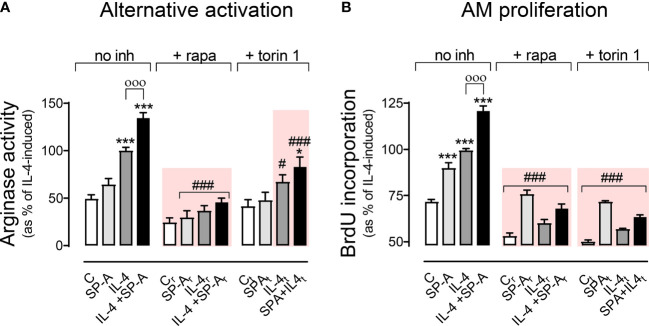
Effect of pharmacological inhibition of mTORC1 on arginase activity and AM proliferation. Purified rat AMs were pretreated with 20 nM **(A)** or 5 nM **(B)** rapamycin, 100 nM **(A)** or 25nM **(B)** torin 1, or vehicle (DMSO) for one hour. Cells were then stimulated with or without IL-4 and/or SP-A and arginase activity **(A)** and AM proliferation **(B)** were measured. The results are presented as means (± SEM) from three different AM cultures with at least three biological replicates. ANOVA followed by the Bonferroni multiple-comparison test was used. *p < 0.05 and ***p < 0.001 when compared with untreated macrophages. ^ооо^ *p* < 0.001 when SP-A+IL-4-treated macrophages were compared with IL-4-treated macrophages. ^#^ *p* < 0.05 and ^###^ *p* < 0.001 when the effect of either rapamycin or torin1 is compared with the same sample without inhibitor.

**Figure 5A** shows that mTORC1 was involved in IL-4- and IL-4+SP-A-dependent alternative activation (synergistic effect), which was sensitive to torin and rapamycin. SP-A alone did not induce a significant increase in arginase activity. Regarding proliferation (**Figure 5B**), IL-4 and IL-4+SP-A (additive effect) induced proliferation dependent on mTORC1 (torin and rapamycin-sensitive). Taken together, these results suggest that the combined effects of IL-4 and SP-A on the mTORC1 and GSK3 branches of Akt signaling likely contribute to increased proliferation, and alternative activation of AMs.

### SP-A Enhancement of IL-4-Dependent Macrophage Alternative Activation Also Requires PKCζ Activation

**Figure 6:**
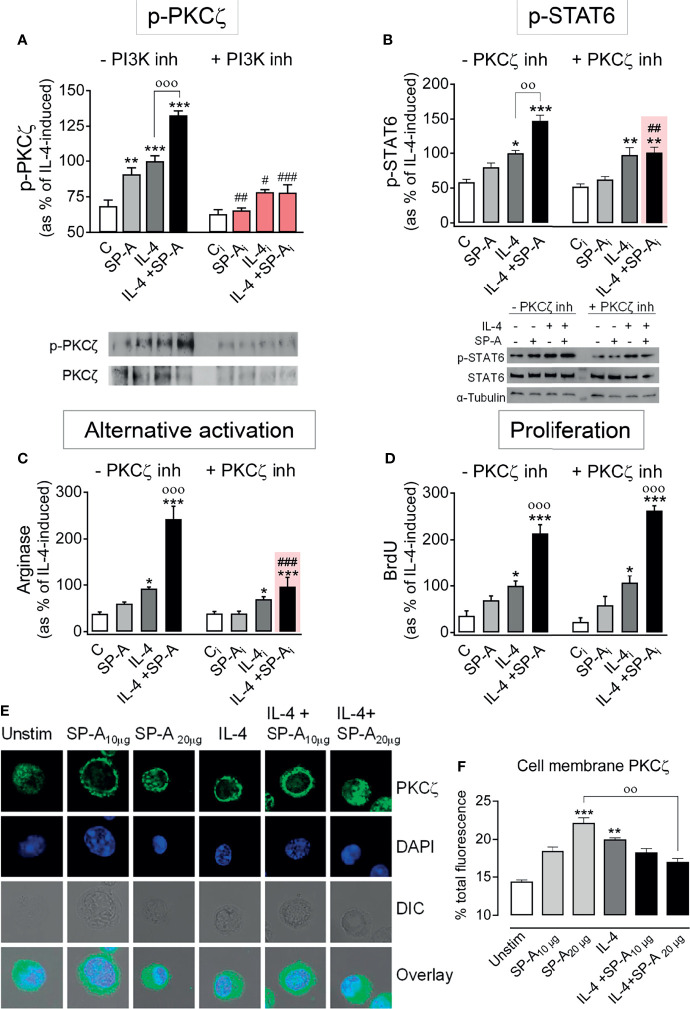
SP-A enhancement of IL-4-induced p-STAT6 and arginase activity is mediated by PKCζ. **(A)** Purified rat AMs were pretreated with 50 nM LY294002 (PI3K inhibitor) or vehicle for two hours. Subsequently, cells were stimulated with or without IL-4 (1 μg/ml) in the presence or absence of SP-A (100 μg/ml). Then p-PKCζ (Thr410/403) relative to total PKCζ was quantified by Western blot. In **(B–D)** cells were pretreated with 30 µM of PKCζ pseudosubstrate inhibitor or vehicle for two hours. Subsequently, cells were stimulated with or without IL-4 (1 μg/ml) and/or SP-A (100 μg/ml). STAT6 phosphorylation relative to total STAT6 **(B)**, arginase activity **(C)**, and proliferation **(D)** of AMs were analyzed. Representative Western blot for p-PKCζ or p-STAT6 are shown below each graph. The results are presented as means (± SEM) from three different AM cultures with at least three biological replicates. **(E)** PKCζ plasma membrane localization after IL-4 and/or SP-A stimulation. Representative IF for PKCζ localization in AMs from SP-A^-/-^ mice left untreated or treated with IL-4 (0.5 μg/ml) in the absence or presence of SP-A (10-20 μg/ml). Confocal images shown are representative of three independent experiments with similar results. Upper panels show PKCζ staining, middle panels show DAPI counterstaining of cell nuclei and differential interference contrast (DIC), lower panels show overlay. **(F)** Quantification of pixel density of cell-membrane PKCζ from total PKCζ values. Data are expressed as percentage ± SEM of three independent experiments with at least 20 cells per condition. Results were statistically analyzed by one-way ANOVA followed by the Bonferroni multiple-comparison test. *p < 0.05, **p < 0.01, and ***p < 0.001 when compared with untreated macrophages. ^оо^ *p* < 0.01 and ^ооо^ *p* < 0.001 when SP-A+IL-4-treated macrophages were compared with IL-4 or SP-A-treated macrophages. ^#^p < 0.05, *^##^p* < 0.01 and ^###^ *p* < 0.001 when the effect of PI3K or PKCζ inhibitor is compared with the same sample without inhibitor.

Atypical protein kinase Cζ (PKCζ) is another protein kinase activated through binding to phosphatidylinositol (3–5)trisphosphate in the plasma membrane and subsequent phosphorylation by PDK1. PKCζ-deficient T cells show reduced STAT6 phosphorylation in response to IL-4 stimulation (27). Accordingly, we analyzed the phosphorylation of PKCζ in alveolar macrophages stimulated with IL-4 and/or SP-A. **Figure 6A** shows that both SP-A and IL-4 induced PKCζ phosphorylation at Thr410, which was significantly increased when alveolar macrophages were stimulated by both factors. The PI3K inhibitor abrogated PKCζ phosphorylation induced by SP-A, IL-4, or SP-A+IL-4, confirming that this process depends on PI3K activation. We hypothesized that SP-A should activate the PI3K/PKCζ axis to enhance IL-4-dependent STAT6 phosphorylation and alternative activation of macrophages, since the SP-A-induced increase in STAT6 phosphorylation was blocked by the PI3K inhibitor (**Figure 2A**).

**Figure 6B** shows that PKCζ inhibition with the PKCζ pseudosubstrate prevented the increase of STAT6 phosphorylation induced by IL-4+SP-A, but not the increase induced by IL-4 alone. As a control experiment, we verified whether Akt was involved in the activation of STAT6 caused by IL-4 or IL-4+SP-A. **Supplementary Figure 3** shows that the production of p-STAT6 by AMs stimulated with IL-4 or IL-4+SP-A did not change in the presence of Akt inhibitor VIII.

Next, we studied the role of PKCζ in SP-A-mediated increase of alternative activation and proliferation of AMs stimulated with IL-4. We found that the PKCζ pseudosubstrate suppressed SP-A enhancement of IL-4-stimulated alternative activation (**Figure 6C**) but not IL-4- and IL-4+SP-A-induced macrophage proliferation (**Figure 6D**). Inhibition of PKCζ kinase activity did not modify IL-4-induced STAT6 phosphorylation (**Figure 6B**) or arginase activity (**Figure 6C**). Together, these results demonstrate that SP-A enhances IL-4-induced alternative activation of alveolar macrophages by sustaining a PI3K/PKCζ/STAT6 signaling axis.

To gain more insight into the functional consequences of cooperative PKCζ phosphorylation by IL-4 and SP-A (**Figure 6A**) for SP-A-mediated enhancement of IL-4-dependent STAT6 activation (**Figure 6B**), we analyzed translocation of PKCζ to the plasma membrane by confocal microscopy of AMs isolated from SP-A-deficient mice, stimulated with IL-4, SP-A, or both (**Figures 6E, F**). We found that IL-4 and SP-A significantly increased PKCζ translocation to the membrane. However, PKCζ appeared to redistribute to endosomes or the cytosol when AMs were stimulated with both agonists (**Figures 6E, F**). It is possible that IL-4+SP-A induces a faster translocation of PKCζ to the plasma membrane than that induced by IL-4 or SP-A alone, so we were unable to detect significant changes at selected times in our experiments. However, our results indicate changes in the cellular location of PKCζ when both agonists are present. On the other hand, it is known that following IL-4 stimulation, activated IL-4 receptors are preferentially associated with cortical endosomes, and there is a link between endocytosis and IL-4R signal transduction (28–30). To know whether SP-A, IL-4, or both agonists increase redistribution of PKCζ to cortical/early endosomes, we determined co-localization of PKCζ and early endosome antigen 1 (EEA1) marker in AMs stimulated with or without these agonists. We found that SP-A, IL-4, and IL-4+SP-A significantly increased PKCζ/EEA1 co-localization, confirming redistribution of PKCζ to early endosomal membranes (**Supplementary Figure 4**).

### SP-A and IL-4 Synergistically Increase Mitochondrial Respiration and Glycolysis in AMs

Metabolic changes in macrophages support and are closely related to the activated macrophage phenotype (31, 32). An increase in both glycolysis and mitochondrial respiration occurs in alternative activation of macrophages. mTORC1 and mTORC2 stimulate glycolysis, mitochondrial function, and induce an anabolic response to promote proliferation and survival (13, 31). Since we have shown that IL-4 and SP-A activate mTORC1 and mTORC2, our aim was to analyze whether the metabolic profile of macrophages stimulated with these agonists is affected. Therefore, we investigate the glycolysis rate and mitochondrial respiration of alveolar stimulated macrophages cultured in RPMI containing 5 mM glucose and 2 mM glutamine, using the mitochondrial stress test (Seahorse XFe24). Measurements of oxygen consumption rate (OCR) were achieved by the combined use of oligomycin (inhibitor of mitochondrial ATP synthase), FCCP (protonophore that uncouples mitochondrial respiration from ATP synthesis), and rotenone plus antimycin A (which blocks mitochondrial respiration by inhibiting complexes I and III, respectively), as previously described (33).

**Figure 7:**
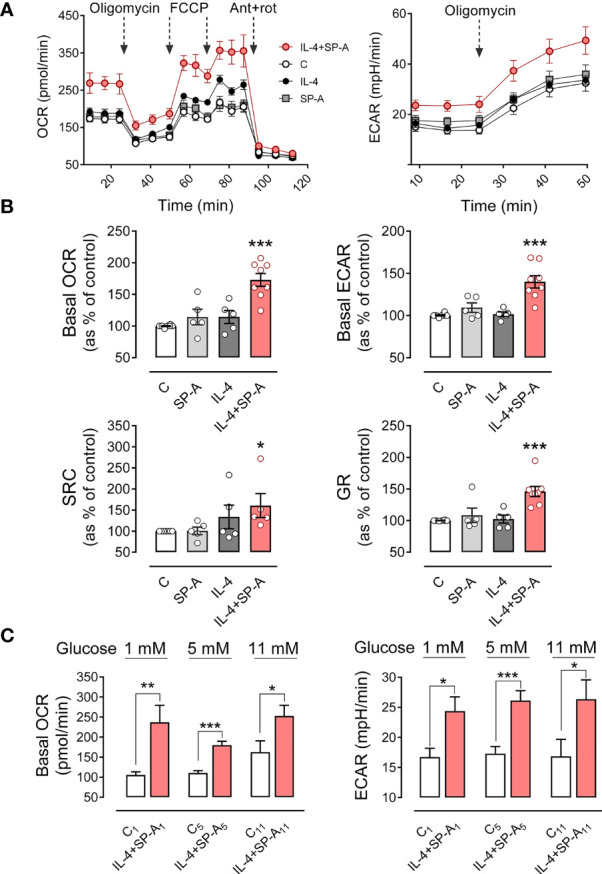
SP-A and IL-4 synergistically increase mitochondrial respiration and glycolysis in AMs. The effect of IL-4 (1μg/ml) and/or SP-A (100 μg/ml) on the oxygen consumption rate (OCR) and the extracellular acidification rate (ECAR) in AM cultures was measured using Seahorse XFe24. **(A)** (left) Alveolar macrophages were treated sequentially with 1 μM oligomycin (ATP synthase inhibitor), 1 μM FCCP (uncoupler of oxidative phosphorylation in mitochondria), and 1 μM rotenone/1 μM antimycin A (inhibitors of mitochondrial complex I and III, respectively) to measure OCR under basal conditions and under mitochondrial stress. (Right) Glycolysis, measured as ECAR, in basal conditions and with oligomycin treatment to evaluate glycolysis intensification in response to mitochondrial inhibition. **(B)** Bar graphs to quantify the effect of IL-4 and/or SP-A on (left) basal OCR and spare respiratory capacity and (right) basal ECAR and glycolytic reserve (increase of ECAR after oligomycin addition). **(C)** Effect of glucose concentration in the culture medium on basal mitochondrial respiration and glycolysis induced by IL-4+SP-A. Data are mean ± SEM from five to eight independent experiments with three to five biological replicates. ANOVA followed by the Bonferroni multiple-comparison test **(A, B)** or Student’s t-test **(C)** was used. *p < 0.05, **p < 0.01, and ***p < 0.001 when compared with untreated macrophages.

Alveolar macrophages stimulated with IL-4 and SP-A exhibited significantly higher basal OCR than macrophages not stimulated or stimulated with each agonist separately. Furthermore, macrophages stimulated with IL-4+SP-A showed a higher level of oligomycin-sensitive respiration, which would be associated with a higher production of ATP in the mitochondria. Respiratory capacity after administration of FCCP was also significantly higher in macrophages stimulated with IL-4+SP-A compared to macrophages not stimulated or stimulated separately with each agonist (**Figures 7A, B**).

The rate of glycolysis, estimated from ECAR values (a proxy for the rate of lactate formation), was also significantly higher in macrophages stimulated with IL-4+SP-A than in those unstimulated or stimulated with IL-4 or SP-A alone. Furthermore, glycolytic capacity, determined after inhibition of mitochondrial ATP synthesis by oligomycin, was also significantly higher in macrophages stimulated with IL-4+SP-A than in those unstimulated or stimulated separately with IL-4 or SP-A. The ability of AMs (stimulated and unstimulated) to induce glycolysis after inhibition of mitochondrial ATP production by oligomycin indicated that alveolar macrophages are not entirely dependent on mitochondria for cellular energy, despite the fact that their natural energy source *in vivo* must be oxidative degradation of surfactant phospholipids (32) (**Figures 7A, B**).

All these parameters indicate that IL-4 and SP-A synergically increased the metabolic activity of alveolar macrophages. As a control, the glucose transport was measured by determining the uptake of the fluorescently labeled deoxyglucose analog 2-NBDG (25) by AMs in the presence or absence of SP-A, IL-4, and both. No differences in glucose transport were observed between stimulated and unstimulated macrophages (**Supplementary Figure 5**), suggesting that there is no upregulation of primary glucose transporter in macrophages (GLUT1) (34) by IL-4.

The level of glucose in alveolar fluid is strongly regulated and is about 12 times lower than the blood glucose concentration (32, 35). Since AMs are exposed *in vivo* to a unique environment with minimal glucose availability, the bioenergetics profile of these cells was also performed in RPMI containing either very low (1mM) or high (11 mM) glucose concentrations. We found similar results regarding the synergistic action of IL-4 and SP-A on mitochondrial respiration and glycolysis (**Figure 7C**), although the increment in mitochondrial respiration of AMs stimulated with IL-4+SP-A decreased with increasing glucose concentration (**Figure 7C**).

Importantly, IL-4 alone did not induce any increase in mitochondrial respiration or glycolysis at glucose concentrations of 1 or 5 mM. However, at a higher glucose concentration of 11 mM, IL-4 alone significantly increased basal OCR and showed a tendency to increase basal ECAR. Both values were further increased in the presence of IL-4+SP-A, confirming that SP-A significantly boosted IL-4-mediated actions in macrophages (**Supplementary Figure 6A**). The bioenergetics profile of cells, defined as the ratio between basal OCR and basal ECAR, was measured in unstimulated and IL-4+SP-A-stimulated macrophages at various glucose concentrations. **Supplementary Figure 6B** shows that the OCR/ECAR ratio in IL-4+SP-A-stimulated compared to unstimulated macrophages was significantly higher at glucose concentrations of 1 mM, but not at glucose concentrations of 5 or 11 mM, indicating that, at low glucose concentrations, IL-4+SP-A notably increased mitochondrial respiration (OCR) for energy production, although macrophages also showed increased anaerobic glycolysis (ECAR) (**Figure 6C**).

## Discussion

Alveolar macrophages are tissue-resident immune cells that colonize the lung around the time of birth and can self-maintain for extended periods in an adult organism. Due to their localization in the airspace of the alveoli, these cells continuously patrol the alveoli and maintain alveolar homeostasis by efficiently removing pathogens, apoptotic cells, and pulmonary surfactant. Under inflammatory conditions such as active bacterial or viral infections, TLR signals and IFN-γ initiate a functional switch in AMs to a pro-inflammatory phenotype. Then, following stimulation with type 2 cytokines (IL-4/IL-3), the macrophage activation state changes to a tissue repair phenotype M(IL-4) (2, 11, 36). This process is driven and amplified by pulmonary surfactant protein A and the uptake of apoptotic cells (4, 37), and it is important to restore tissue homeostasis after injury. However, it can lead to allergy and fibrosis if not properly regulated (1, 2, 11).

**Figure 8:**
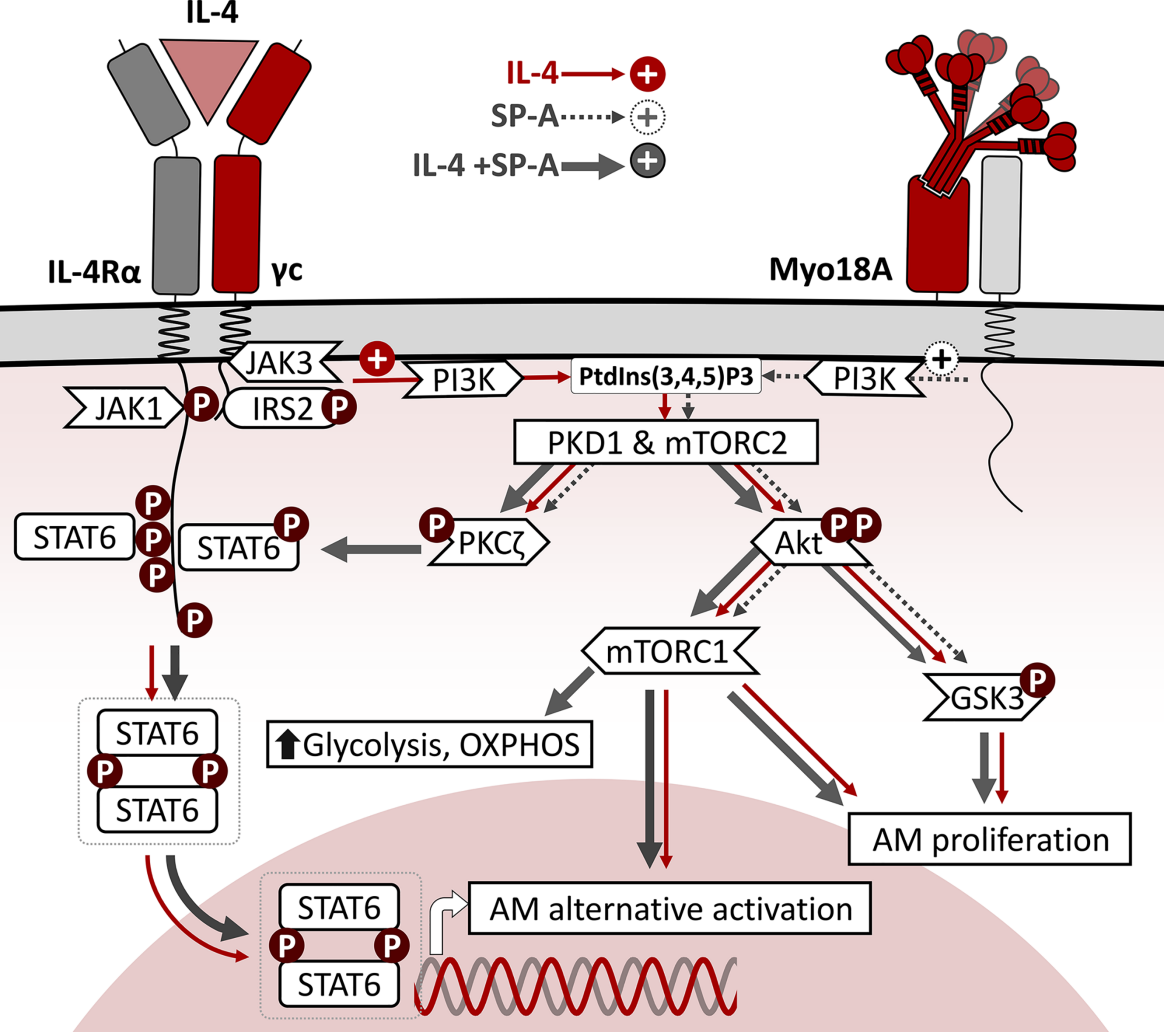
Schematic representation of intracellular signaling pathways of IL-4, SP-A, and IL-4+SP-A in alveolar macrophages. The binding of IL-4 to the ectodomains of IL-4Rα and γc chains results in phosphorylation of critical tyrosine residues in IL-4Rα cytosolic domain, which serves as docking sites for SH domains of intracellular signaling molecules such as STAT6 and IRS2. Once STAT6 is activated by phosphorylation, they homodimerize *via* their Src homology 2 domains and translocate to the nucleus, where they bind to specific DNA sequences that induce expression of genes associated with alternative activation. In parallel, efficient tyrosine phosphorylation of IRS2 triggers the PI3K/Akt pathway, associated to the plasma and/or endosomal membranes. This signaling pathway activates cell proliferation and AM alternative activation. SP-A, *via* the Myo18A receptor, activates PI3K, Akt, mTORC1, and PKCζ, and inactivates GSK3α/β by phosphorylation, but cannot activate arginase-1 activity or AM proliferation. The combined effects of IL-4 and SP-A on the mTORC1 and GSK3 branches of PI3K-Akt signaling contribute to increased AM proliferation and alternative activation. Consistent with mTORC1 activation, SP-A+IL-4 synergistically increased mitochondrial respiration and glycolysis in AMs. In addition, SP-A+IL-4-mediated PKCζ signaling seems to intersect PI3K activation with STAT6 phosphorylation, increasing alternative activation of IL-4-stimulated macrophages.

In this study we focus on the alternative activation and cell cycling of alveolar macrophages stimulated by IL-4 alone and in combination with SP-A, at SP-A concentrations within the ranges found in the alveolar fluid of rat and human lungs (38, 39). We analyze the signaling pathways by which IL-4 and SP-A synergistically increase arginase-1 activity and cell proliferation (4) and determine the influence of these two factors on the metabolic profile of AMs. **Figure 8** summarizes the intracellular signaling pathways of IL-4, SP-A, and IL-4+SP-A analyzed in this study. By examining the phosphorylation of key transcription factors and regulatory enzymes and using pharmacological inhibitors, we found that SP-A activates coordinated PI3K-dependent signaling pathways that amplify the actions of IL-4 in alveolar macrophages. SP-A alone activates Akt, mTORC1, and PKCζ, and inactivates GSK3α/β by phosphorylation, but cannot activate arginase-1 activity or AM proliferation. Our results indicate that the combined effects of IL-4 and SP-A on the mTORC1 and GSK3 branches of the PI3K-Akt signaling axis contribute to increased proliferation and alternative activation of AMs. On the other hand, IL-4+SP-A-mediated PKCζ signaling appears to intersect PI3K activation with STAT6 phosphorylation, which increases alternative activation of IL-4-stimulated macrophages (**Figure 8**).

PKCζ is highly expressed in the lung (40), where it plays a critical role in several biological processes, including cell polarity and signaling. Regarding PKCζ signaling, in this study we found that IL-4 and SP-A synergistically increase (i) phosphorylation of PKCζ at Thr^410/403^ in a PI3K-dependent manner; ii) phosphorylation of STAT6 in a PI3K- and PKCζ-dependent manner; and iii) alternative activation, but not proliferation, of AMs in a PI3K- and PKCζ-dependent manner. Macrophages stimulated with SP-A alone show increased PKCζ phosphorylation, as previously reported (17), but not STAT6 phosphorylation. On the other hand, macrophages stimulated with IL-4 alone show increased PKCζ phosphorylation, but STAT6 phosphorylation and macrophage alternative activation were not affected by pharmacological inhibition of PKCζ. These results contrast with those obtained for lymphocytes (27, 41, 42), where the loss of PKCζ impaired Jak1 activation, STAT6 phosphorylation, and secretion of Th2 cytokines *in vitro* and *in vivo*, indicating that PKCζ is necessary for Th2 differentiation (27, 41, 42). PKCζ^-/-^ mice show dramatic inhibition of ovalbumin-induced allergic airway disease (40), strongly suggesting that PKCζ is critical for IL-4 signaling in the lung. In this study we show that SP-A, *via* PKCζ, increased IL-4-dependent phosphorylation of STAT6, essential for downstream targets of IL-4 signaling.

The catalytic potential of PKCζ, and other PKCs, is dictated by ‘priming’ phosphorylation in their catalytic domains, which are largely conserved (40, 43). But, as in other members of the PKC family, phosphorylated enzymes are maintained in a self-inhibited conformation through the interaction of their inhibitory pseudosubstrate domain with their catalytic domain (40, 43). Activation of PKCζ and other PKCs involves their binding to the membrane surface (43). For PKCζ, membrane binding occurs through two mechanisms that work in tandem: i) binding of the scaffold protein Par6 (bound to the N-terminal PB1 domain of PKCζ) to CDC42/Crumbs (bound to membrane) (43, 44), and ii) electrostatic interaction between the polybasic region of the PKCζ inhibitory pseudosubstrate with anionic phospholipids (PI4P and PI4,5P2) present in the plasma membrane and endosomal membranes (44). Binding of PKCζ to the membrane surface facilitates conformational changes in the protein that lead to dissociation of the pseudosubstrate from the catalytic domain and trigger PKCζ kinase activity (43, 44). In this study, we analyzed the effect of SP-A and/or IL-4 on the recruitment of PKCζ to the plasma membrane and endosomal membranes using confocal microscopy. Macrophages stimulated with IL-4 or SP-A alone show a significant increase in translocation of PKCζ to the plasma membrane and cortical/early endosomes. Interestingly, when macrophages were stimulated with IL-4+SP-A, PKCζ appeared to redistribute to early endosomes or the cytosol, suggesting a rapid alteration of the cell location of PKCζ in the presence of both agonists. It is possible that the presence of both agonists induces a faster internalization of the IL-4Rα receptor (28–30) and the movement of PKCζ from the plasma membrane to early endosomes. The presence of SP-A would improve the phagocytic and endocytic capacity of AMs (18, 45). SP-A uptake by AMs precede the initiation of SP-A signaling (18). It remains to be determined whether SP-A could affect other factors that regulate the activity and location of PKCζ. A potential candidate is the soluble lipid mediator sphingosine-1-phosphate (S1P), which is an allosteric activator of PKCζ catalytic activity (46).

Following IL-4 stimulation, IL-4Rα/γc is thought to be internalized, leading to increased receptor density in endosomal membranes (28, 29). Whether IL-4Rα/γc endocytosis is essential for downstream signaling transduction is still debated, but Bai et al. (30) recently demonstrated that endocytosis of IL-4Rα/γc is essential for Akt activation, but not STAT6 activation. Macrophage stimulation by both agonists, IL-4 and SP-A, converges on activation of the PI3K-Akt signaling pathway. In this study, we found that an increase in Akt phosphorylation was elicited by SP-A, IL-4 or SP-A+IL-4 macrophage stimulation and was completely suppressed by PI3K inhibitor and torin (inhibitor of mTORC1 and 2) but not by rapamycin (specific inhibitor of mTORC1).

The phosphorylation of GSK3, FOXO3, and the activation of mTORC1, which are downstream targets of Akt (26), were also evaluated. Consistent with the activation of Akt, we show that SP-A, IL-4, and IL-4+SP-A significantly increased phosphorylation of GSK3α and β subunits, which results in the inhibition of their kinase activity. GSK3 is a soluble monomeric enzyme that shows constitutively high basal kinase activity in resting cells, which phosphorylates and inactivates several proteins involved in proliferation (e.g., the transcription factors c-Myc and c-JUN), survival (e.g., MCL-1 that belongs to the Bcl-2 family), or metabolism (e.g., glycogen synthase) (26, 47, 48). Akt inhibits GSK3 by phosphorylation of serine Ser21 at GSK3α and Ser9 at GSK3β, activating cell metabolism, proliferation, and survival (26, 47, 48). This suggests that GSK3 phosphorylation induced by IL-4 or IL-4+SP-A macrophage stimulation might be involved in macrophage proliferation since Akt inhibitor VIII drastically suppressed macrophage proliferation induced by IL-4 alone and IL-4+SP-A as well as the phosphorylation of GSK3. Evidence supports that GSK3 phosphorylation is involved in the resolution of inflammation, tissue repair, and macrophage proliferation (47, 48). On the contrary, active GSK3 suppresses STAT3/6 phosphorylation to promote classical macrophage activation (49). Regarding FoxO3a, we did not find a significant effect of SP-A and/or IL-4 on FoxO3a phosphorylation, a transcription factor also involved in cell proliferation (26).

Another consequence of Akt activation in macrophages stimulated with SP-A, IL-4, and IL-4+SP-A was a significant increase in mTORC1 activity. The activity of mTORC1, measured by phosphorylation of 4E-BP1, was completely inhibited by rapamycin and torin1. The ability of IL-4 and IL-4+SP-A to increase arginase activity and macrophage proliferation was sensitive to torin and rapamycin, and therefore, dependent on mTORC1 and mTORC2. According to these results, the PI3K/Akt/mTORC1 axis was reported to be involved in IL-4-induced alternative activation and proliferation of BMDM (13, 14). Increased alternative macrophage activation is reported to be mediated by increased acetylation of histones that controls upregulation of alternative activation genes (*Arg*1 and *Retnla*) and genes involved in proliferation, DNA replication (13, 50), and glycolysis (50, 51). Histone acetylation depends on the levels of acetyl-CoA in the cytosol derived from glucose and produced by the cytosolic enzyme ATP citrate lyase (Acly) (51). The Akt-mTORC1 pathway is involved in the regulation of Acly: Akt phosphorylates and activates Acly (52), and mTORC1 increases the expression of Acly in macrophages (13). Cultured Acly-deficient macrophages show decreased IL-4 response, which may be explained by lower acetylation of histone 3 lysine 27 (53).

The metabolic profile of *in vitro* cultured alveolar macrophages, stimulated with IL-4+SP-A but not with SP-A or IL-4 alone, is consistent with IL-4+SP-A-induced cooperative activation of mTORC1 and mTORC2, which play a prominent role in increasing glycolysis and anabolic processes for cell growth and proliferation (31, 54, 55). We found that alveolar macrophages exhibited significantly higher mitochondrial respiration (basal OCR values) when cells were stimulated with both IL-4 and SP-A but not when stimulated with each agonist separately at 5 mM glucose. Likewise, the rate of anaerobic glycolysis (ECAR values, as a proxy for the rate of lactate formation) was significantly higher in macrophages stimulated with IL-4+SP-A than in those not stimulated or stimulated with IL-4 or SP-A separately. Therefore, IL-4 and SP-A act synergistically to increase mitochondrial respiration (required to produce energy for biosynthetic reactions and proliferation) and the glycolysis pathway, which provides intermediate metabolites for the pentose phosphate pathway, glycosylation reactions and synthesis of key biomass constituents, including serine, glycine, glycerol-3-P, and acetyl-CoA for lipid synthesis (56).

An important limitation of these studies is that *in vitro* studies cannot reproduce the unique environment in which AMs reside, which is rich in lipids but very poor in glucose and amino acids (32, 35). Since glucose levels can influence the expression of Acly (57), the metabolic profile of AMs was also carried out with RPMI supplemented with glutamine and very low (1mM) or high (11 mM) glucose concentrations. Regardless of glucose concentration, IL-4 and SP-A acted synergistically to increase mitochondrial respiration and glycolysis, indicating the importance of SP-A in driving IL-4-mediated metabolic reprogramming in macrophages. Interestingly, at the highest glucose concentration (11 mM), but not at glucose concentrations of 5 or 1 mM, IL-4 alone significantly increased basal OCR and showed a tendency to increase basal ECAR, indicating the influence of the medium’s glucose concentration on the response of AMs to IL-4.

In summary, this study provides a novel mechanism for the action of SP-A on IL-4-dependent signal transduction underlying macrophage repair responses (**Figure 8**). Although IL-4-activated macrophages have recently emerged as important players in homeostatic processes, chronic respiratory diseases such as fibrosis, asthma, and allergy are associated with a dysregulated type 2 response (1, 2, 11). The pro-M(IL-4) effects of SP-A reported here and in (4) contrast with reports that associate SP-A with protection in asthma and fibrosis (58–60). However, in addition to promoting IL-4-dependent activation and proliferation of alveolar macrophages, the anti-inflammatory and protective properties of SP-A (7–9, 60) may suppress the strong inflammatory responses that are responsible for more severe asthma and fibrosis. Type 2 cytokines are also important regulators of type 1- and TH17-driven inflammatory responses. Thus, several studies have revealed that blocking type 2 cytokines can dysregulate this cross-regulatory mechanism and promote type 1- and TH17-driven inflammation (1, 61). However, dual blockade of IL-13 and IFNγ leads to a marked reduction in fibrosis and eliminates the type 1 rebound inflammation and associated damage (62). Moreover, inhibition of PI3K/Akt/mTOR and TLR4/MyD88/NF-κB signaling with targeted molecules can attenuate pathological mechanisms of asthma and play an important role in protecting airways against allergic response and inflammation pathology (63). The signaling events that drive the SP-A-dependent amplification of IL-4 effects on alveolar macrophages may aid in the development of new approaches to control lung diseases caused by exaggerated repair responses.

## Data Availability Statement

The raw data supporting the conclusions of this article will be made available by the authors, without undue reservation.

## Ethics Statement

The animal study was reviewed and approved by Local ethics committee of Complutense University of Madrid and Autonomous Community of Madrid (Spain).

## Author Contributions

Conceptualization, BG-F, CM, CS, and CC. Methodology and Investigation, BG-F, CM, CM-F, and CS. Formal analysis, all authors. Writing—original draft preparation, BG-F and CM. Writing—review and editing, CC. Supervision, CC. Project administration, CC. Funding acquisition for this study, CC. All authors read and agreed to the published version of the manuscript.

## Funding

This study was supported by the Spanish Ministry of Science, Innovation and and Universities through Grants SAF2015-65307-R and RTI2018-094355‐B‐I00 to CC.

## Conflict of Interest

The authors declare that the research was conducted in the absence of any commercial or financial relationships that could be construed as a potential conflict of interest.

## Publisher’s Note

All claims expressed in this article are solely those of the authors and do not necessarily represent those of their affiliated organizations, or those of the publisher, the editors and the reviewers. Any product that may be evaluated in this article, or claim that may be made by its manufacturer, is not guaranteed or endorsed by the publisher.
