# Supplementary material for: Signaling Pathways That Mediate Alveolar Macrophage Activation by Surfactant Protein A and IL-4
Source: Front Immunol. 2022 Apr 4;13:860262. doi: 10.3389/fimmu.2022.860262 (PMC9014242; doi:10.3389/fimmu.2022.860262)
Supplement: Supplementary file 1 [file DataSheet_1.pdf]

Supplementary Material

Signaling pathways that mediate alveolar macrophage activation by surfactant protein A and IL-4

Belén García-Fojeda[1]†, Carlos M. Minutti[1]†*, Carlos Montero-Fernández[1], Cordula Stamme[2,3] and Cristina Casals[1]*

[1]Department of Biochemistry and Molecular Biology, Complutense University of Madrid, Madrid, Spain.
[2]Division of Cellular Pneumology, Research Center Borstel, Leibniz Lung Center, Borstel, Germany.
[3]Department of Anesthesiology and Intensive Care, University of Lübeck, Lübeck, Germany.

(†) These authors have contributed equally to this work and are listed in alphabetical order.

***Correspondence:**
Cristina Casals and Carlos M. Minutti.
Department of Biochemistry and Molecular Biology, Faculty of Biology, Complutense University of Madrid, 28040 Madrid, Spain,
Tel.: (34) 913944261,
ccasalsc@ucm.es; carlos.minutti@gmail.com

Keywords: IL-4, surfactant protein A, macrophage alternative activation, proliferation, PI3K-Akt, mTORC1, PKCζ, metabolism**.**

Running Title**: IL-4/SP-A signaling in alveolar macrophages**

Research Topic in Molecular Innate Immunity: Updates on the Role of Surfactant Proteins A and D in Innate Immune Responses

Supplementary Figures

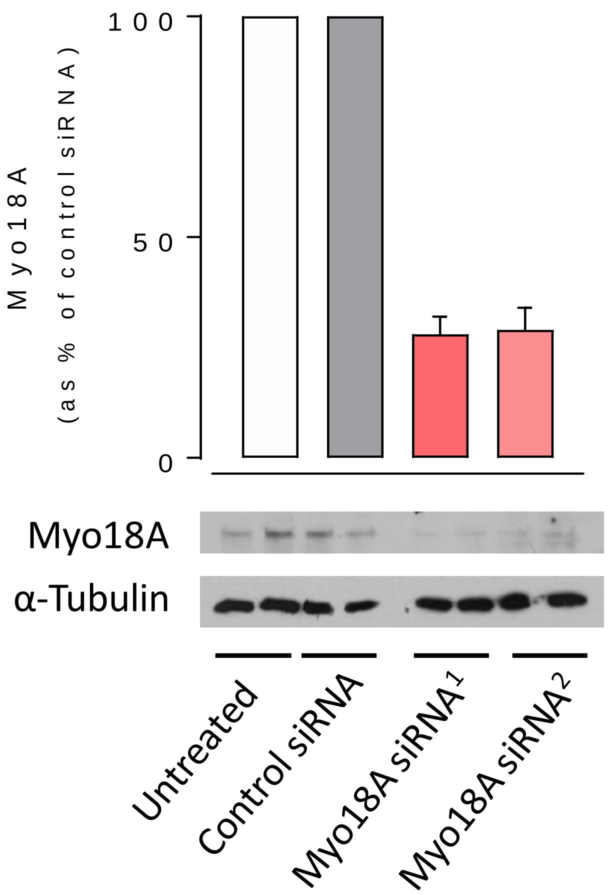

.

Supplementary Figure 1. siRNA-targeted silencing of Myo18A. Purified rat AMs were nucleofected with 100 nM Stealth siRNAs directed against rat Myo18A, designated Myo18A siRNA[1] (RSS322720) and Myo18A siRNA[2] (RSS322721), and GC Stealth siRNA medium was used as control (Control siRNA). 48 hours after nucleofection, Myo18A expression was analyzed by Western blot. Data are the mean ± SEM of three independent experiments run in duplicate. A representative Western blot analysis of Myo18A is shown.

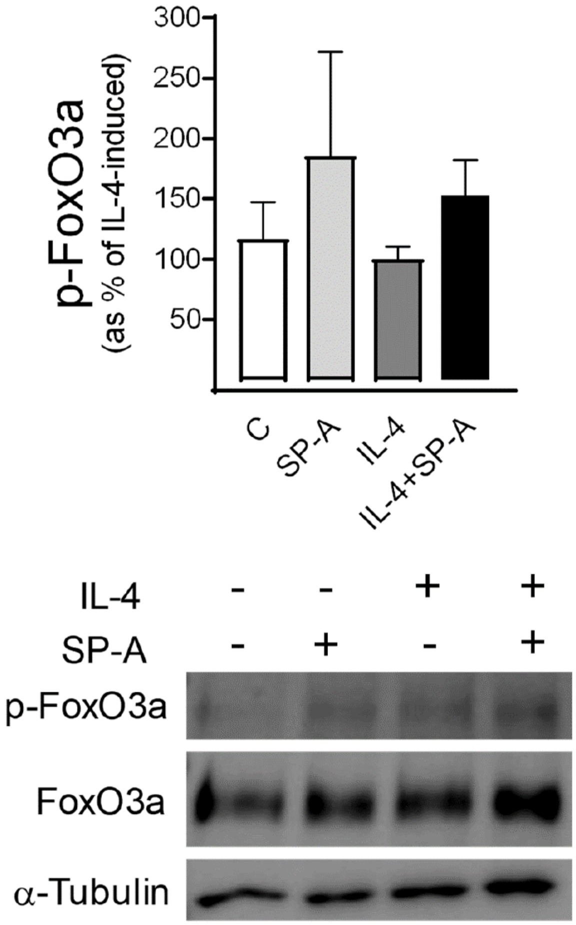

Supplementary Figure 2. Neither SP-A, IL-4, nor IL-4+SP-A enhances phosphorylation of FoxO3a. Purified rat AMs were left unstimulated or stimulated with IL-4 (1 µg/ml) in the presence or absence of SP-A (100 µg/ml) for 45 min. Then, p-FoxO3a (Thr32) was analyzed. A representative Western blot analysis is shown. Data are mean ± SEM from three independent experiments run in triplicate. ANOVA followed by the Bonferroni multiple-comparison test was used.

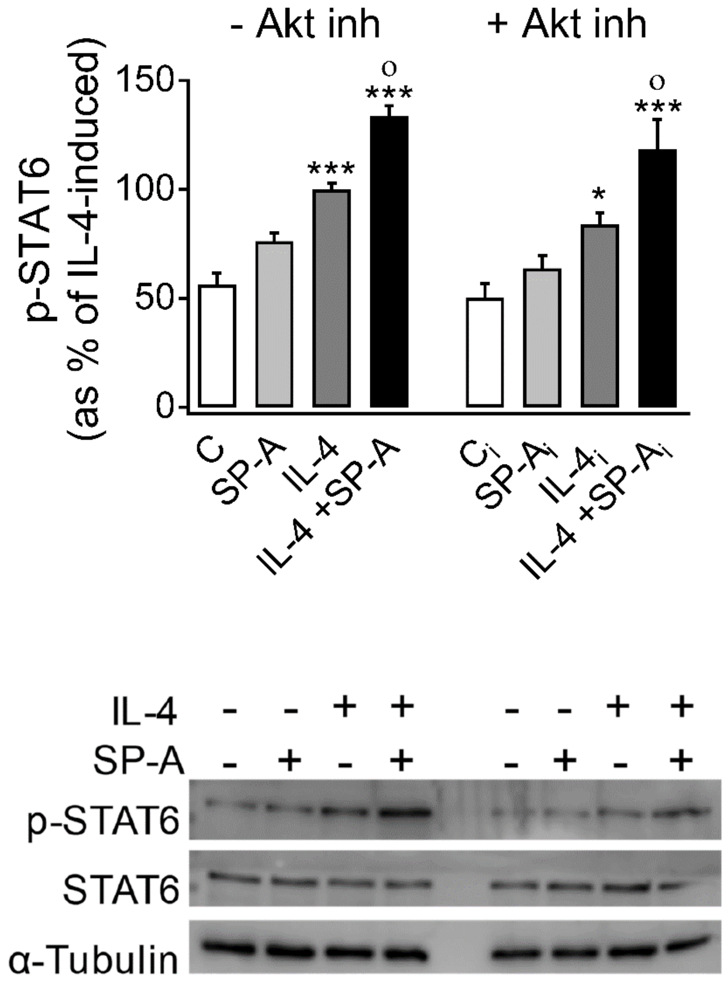

Supplementary Figure 3. Effect of Akt inhibitor on STAT6 phosphorylation. Purified rat AMs were pretreated with 25 nM Akt Inhibitor VIII or vehicle for two hours. Subsequently, cells were stimulated with or without IL-4 (1 μg/mL) and/or SP-A (100 μg/mL), and STAT6 phosphorylation relative to total STAT6 was analyzed by Western blot. A representative Western blot for p-STAT6 is shown. The results are presented as means (± SEM) from three different AM cultures with at least three biological replicates and were statistically analyzed by one-way ANOVA and Bonferroni multiple-comparison test. *$p < 0.05$, **$p < 0.01$, and ***$p < 0.001$ when compared with untreated macrophages; $^{o}p < 0.05$ when SP-A+IL-4-treated macrophages were compared with IL-4 or SP-A-treated macrophages.

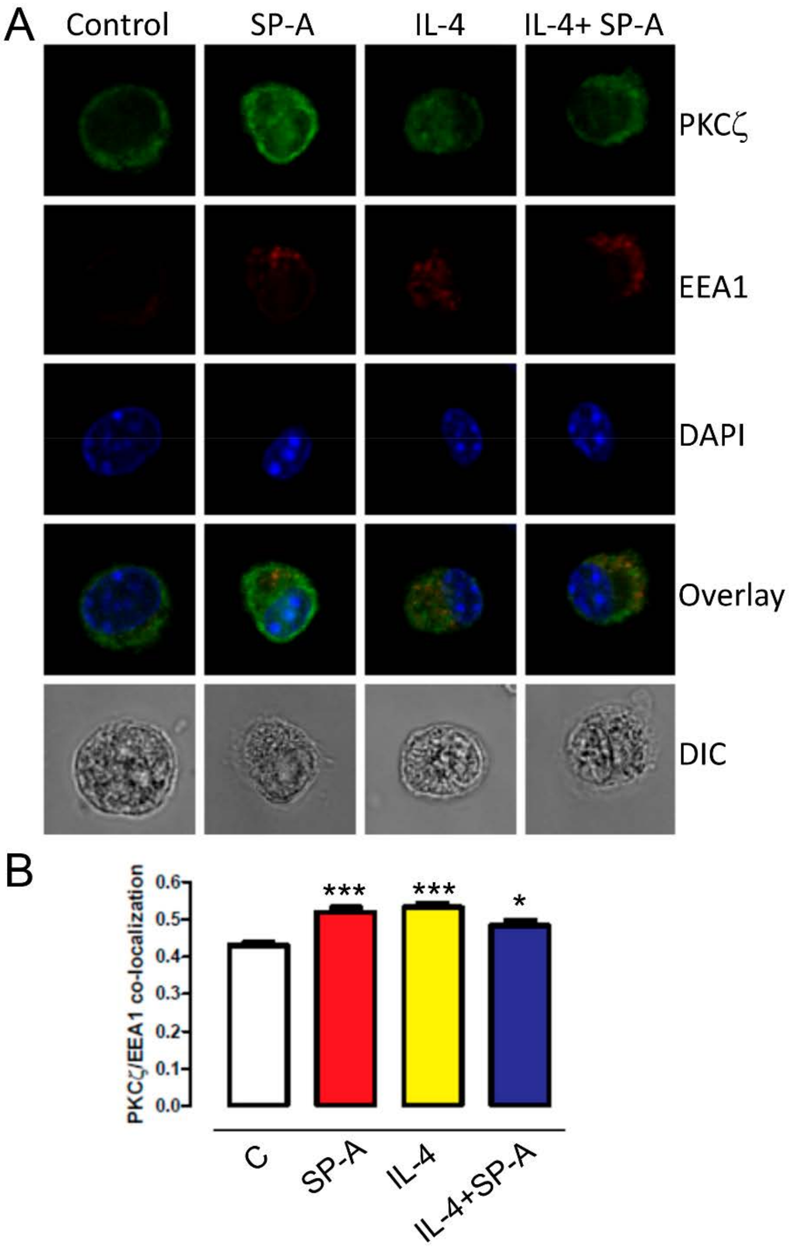

Supplementary Figure 4. PKCζ colocalization with early endosomes after IL-4 and/or SP-A stimulation. (A) Representative IF for PKCζ co-localization in AMs from SP-A$^{-/-}$ mice left untreated or treated with IL-4 (0.5 μg/ml) in the absence or presence of SP-A (20 μg/ml). Upper panels show PKCζ staining; middle panels show EEA1 staining, DAPI counterstaining, and overlay; lower panels show differential interference contrast (DIC). **(B)** Quantification of pixel density of PKCζ/EEA1 co-localization. Data are expressed as mean ± SEM of three independent experiments with at least 30 cells per condition. ANOVA followed by Bonferroni multiple-comparison test was used. *$p < 0.05$, ***$p < 0.001$.

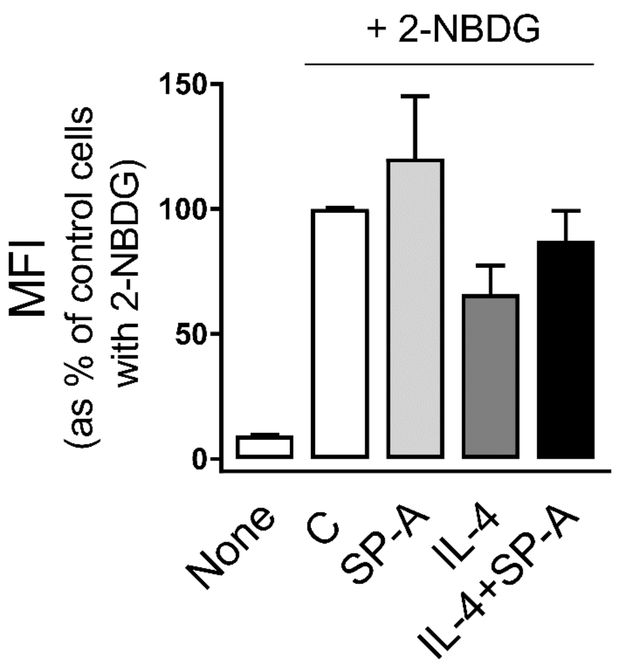

Supplementary Figure 5. Glucose uptake of AMs after 24 h incubation with and without IL-4, SP-A, and IL-4+SP-A. Rat AMs were left unstimulated or stimulated with IL-4 (1 µg/ml) and/or SP-A (100 µg/ml) for 24 h. Then, the deoxyglucose fluorescent analogue 2-NBDG (146 µM) was added for 20 minutes, and cells were washed and analysed by flow cytometry. Mean fluorescence intensity is represented as % of control cells. Data are mean ± SEM from three independent experiments with three biological replicates. ANOVA followed by the Bonferroni multiple-comparison test was used to compare stimulated or unstimulated macrophages in the presence of the fluorescent glucose analogue 2-NBDG.

.

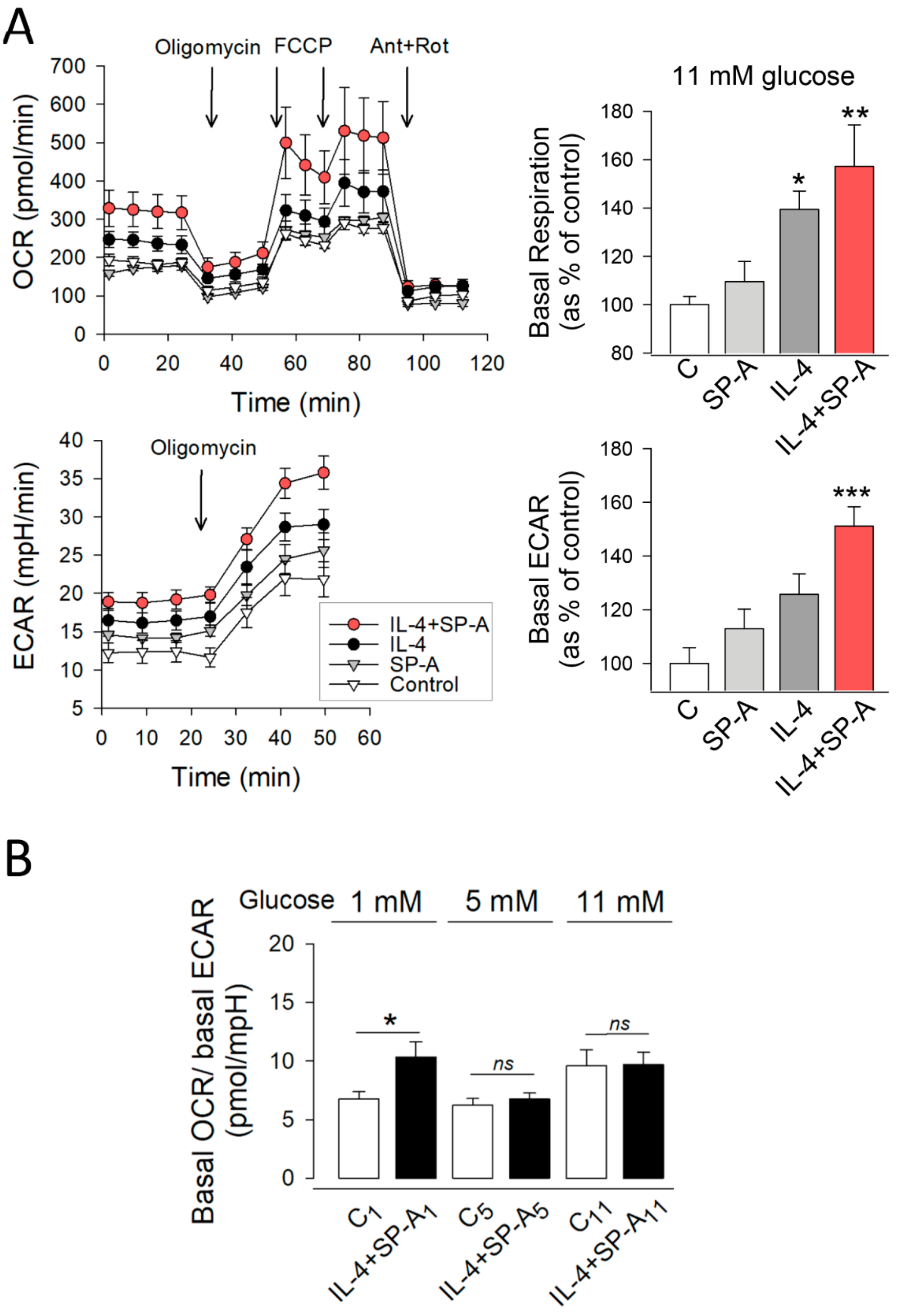

Supplementary Figure 6. Effect of glucose concentration on the metabolic profile of AMs. (A) Rat AMs were grown overnight in RPMI medium containing 11 mM glucose and supplemented with 2 mM glutamine, 5% heat-inactivated FBS, and 100 U/ml of penicillin/streptomycin. Cells were then left unstimulated or stimulated with IL-4 (1 µg/ml) and/or SP-A (100 µg/ml) for 24 h, and both OCR

and ECAR were measured using Seahorse XFe24. Left panels show OCR and ECAR measurements in a representative experiment of three independent assays. Right panels show bar graphs to quantify basal respiration (OCR) and basal ECAR. **(B)** The bioenergetics profile of macrophages (defined as the ratio between basal OCR and basal ECAR) unstimulated and stimulated with IL-4+SP-A in RPMI medium containing 1 mM, 5 mM, or 11 mM glucose. Data are mean ± SEM from three independent experiments with three to four biological replicates. In (A) ANOVA followed by the Bonferroni multiple-comparison test was used. In (B) Student's t-test was used. *$p < 0.05$, **$p < 0.01$, and ***$p < 0.001$ when compared with untreated macrophages.
